# Efficient and High‐Purity Sound Frequency Conversion with a Passive Linear Metasurface

**DOI:** 10.1002/advs.202203482

**Published:** 2022-10-17

**Authors:** Wei Wang, Chengbo Hu, Jincheng Ni, Yujiang Ding, Jingkai Weng, Bin Liang, Cheng‐Wei Qiu, Jian‐Chun Cheng

**Affiliations:** ^1^ Collaborative Innovation Center of Advanced Microstructures and Key Laboratory of Modern Acoustics MOE Institute of Acoustics Department of Physics Nanjing University Nanjing 210093 China; ^2^ Department of Electrical and Computer Engineering National University of Singapore 4 Engineering Drive 3 Singapore 117583 Singapore

**Keywords:** acoustic metasurface, acoustic orbital angular momentum, high‐efficiency frequency conversion, rotational Doppler effect

## Abstract

Despite the significance for wave physics and potential applications, high‐efficiency frequency conversion of low‐frequency waves cannot be achieved with conventional nonlinearity‐based mechanisms with poor mode purity, conversion efficiency, and real‐time reconfigurability of the generated harmonic waves in both optics and acoustics. Rotational Doppler effect provides an intuitive paradigm to shifting the frequency in a linear system which, however, needs a spiral‐phase change upon the wave propagation. Here a rotating passive linear vortex metasurface is numerically and experimentally presented with close‐to‐unity mode purity (>93%) and high conversion efficiency (>65%) in audible sound frequency as low as 3000 Hz. The topological charge of the transmitted sound is almost immune from the rotational speed and transmissivity, demonstrating the mechanical robustness and stability in adjusting the high‐performance frequency conversion in situ. These features enable the researchers to cascade multiple vortex metasurfaces to further enlarge and diversify the extent of sound frequency conversion, which are experimentally verified. This strategy takes a step further toward the freewheeling sound manipulation at acoustic frequency domain, and may have far‐researching impacts in various acoustic communications, signal processing, and contactless detection.

## Graphical abstract

The first theoretical design and experimental demonstration of a new mechanism of amplitude‐independent frequency conversion are presented for airborne sound within a physical distance much smaller than the wavelength by using rotating linear metasurfaces, which break the current limitations in terms of mode purity, conversion efficiency, frequency shift, and device compactness.

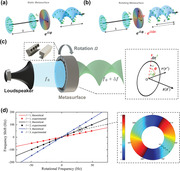

## 1 Introduction

Frequency conversion is of fundamental interest in wave physics and conventionally realized by utilizing nonlinear, up‐/downconversion materials in optics. The most intuitive way of frequency conversion for light is to use the nonlinear process in a strong laser electric field, in which the energy of fundamental wave will be partially converted to high‐order harmonic waves.^[^ ^1^ ^]^ By the Stokes/anti‐Stokes process, photon down/up‐conversion materials can emit a lower/higher energy photon than the absorbed photons.^[^ ^2^, ^3^ ^]^ Despite a rapid progress in the research field, wide applications of frequency conversion in optics are strongly limited because of their overall low efficiencies. In acoustics, inhomogeneity of medium such as pulsating bubbles can significantly enhance the equivalent nonlinearity parameter.^[^ ^4^, ^5^ ^]^ However, the intrinsic dependence of frequency and driving amplitude limits the efficiency of nonlinear frequency conversion and makes it impossible to produce only one harmonic wave for low‐frequency and weak‐amplitude sound.

In the linear regime, frequency conversion can also be observed on the basis of the Doppler effect, which stems from the relative motion of the wave source and receiver. This can be classified into two categories: 1) the translational Doppler effect, which is caused by the translational motion of the receiver or wave source, is extensively used for detecting an object's moving velocity with light or acoustic wave (such as in speed detection); 2) the rotational Doppler effect, which has a relative rotational motion between spiral wave front and receiver and leads to frequency shift, can be used to detect the rotation speed of objects. For electromagnetic waves, it was reported that the rotational Doppler effect arises from the photonic orbital angular momentum (OAM) by vortex beams.^[^ ^6^ ^]^ The vortex beams have a helical phase structure of exp (*ilϕ*) proportional to the azimuthal angle *ϕ* with an unbounded integer *l* referring to the topological charge.^[^ ^7^, ^8^, ^9^, ^10^, ^11^, ^12^, ^13^ ^]^ The OAM of light has grown into a significant research field, giving rise to many developments in optical sensing, micromanipulation, micro/nanofabrication, imaging and microscopy, and optical communications.^[^ ^14^, ^15^, ^16^, ^17^, ^18^ ^]^ In particular, a vortex generator with tunable topological charges is a critical step in the realization of OAM modulation and multiplexing in both optical and acoustic communications.^[^ ^19^, ^20^, ^21^ ^]^ Generally, the vortex generators are designed for specific frequencies, implying that phase distortion or mode crosstalk would be inevitable after the operating frequency is finely altered. On the other hand, the OAM‐frequency division multiplexing for multidimensional high‐capacity information processing requires stable OAM modes at different frequencies, which is not yet accessible by static devices. The rotational Doppler frequency shift for photonic OAM can be given as Δ*ω* = *l*Ω in the scheme with the vortex beam detected by a transducer rotating around the beam axis.^[^ ^22^, ^23^, ^24^, ^25^, ^26^, ^27^, ^28^, ^29^, ^30^, ^31^ ^]^ Also, the backscattering of light carrying OAM on a rough rotating surface has been shown to undergo a rotational Doppler frequency shift.^[^ ^31^ ^]^

Although the longitudinal nature of acoustic waves lacks spin angular momentum, airborne sound can still carry OAM by active and passive devices.^[^ ^7^, ^32^, ^33^ ^]^ In particular, the rotational Doppler frequency shift of sound has been observed acoustically, but needs an incident acoustic vortex beam and a spinning detector, which would be difficult to implement in practice.^[^ ^34^ ^]^ To date, a high‐efficiency and high‐purity frequency conversion of low‐frequency sound in static medium remains challenging.

In the present work, we theoretically propose and experimentally demonstrate a mechanism to overcome the above limitations by a rotation system and a high‐transmittance acoustic OAM metasurface. The phase‐adjustable and high‐transmittance units are periodically arranged along the azimuthal direction, forming a spiral phase distribution for the incident plane wave at working frequency *f*_0_. A rotation system drives the metasurface to rotate steadily at an angular velocity of Ω. In this case, the phase distribution on the transmission surface leads to the rotational Doppler effect, and the transmitted wave can be converted to a new frequency expressed as *f*_0_ + *l*Ω/2*π*. Meanwhile, the high transmittance of the metasurface maintains in the rotation and keeps the conversion effective. We also demonstrate the robustness of the incident wavefront experimentally, meaning that high efficiency and frequency shift can be maintained regardless of the incident wavefront. This enables cascading of the frequency convertor for further enhancing the frequency shifts, which is also verified experimentally on frequency conversion and efficiency.

## 2 Results

**Figure 1:**
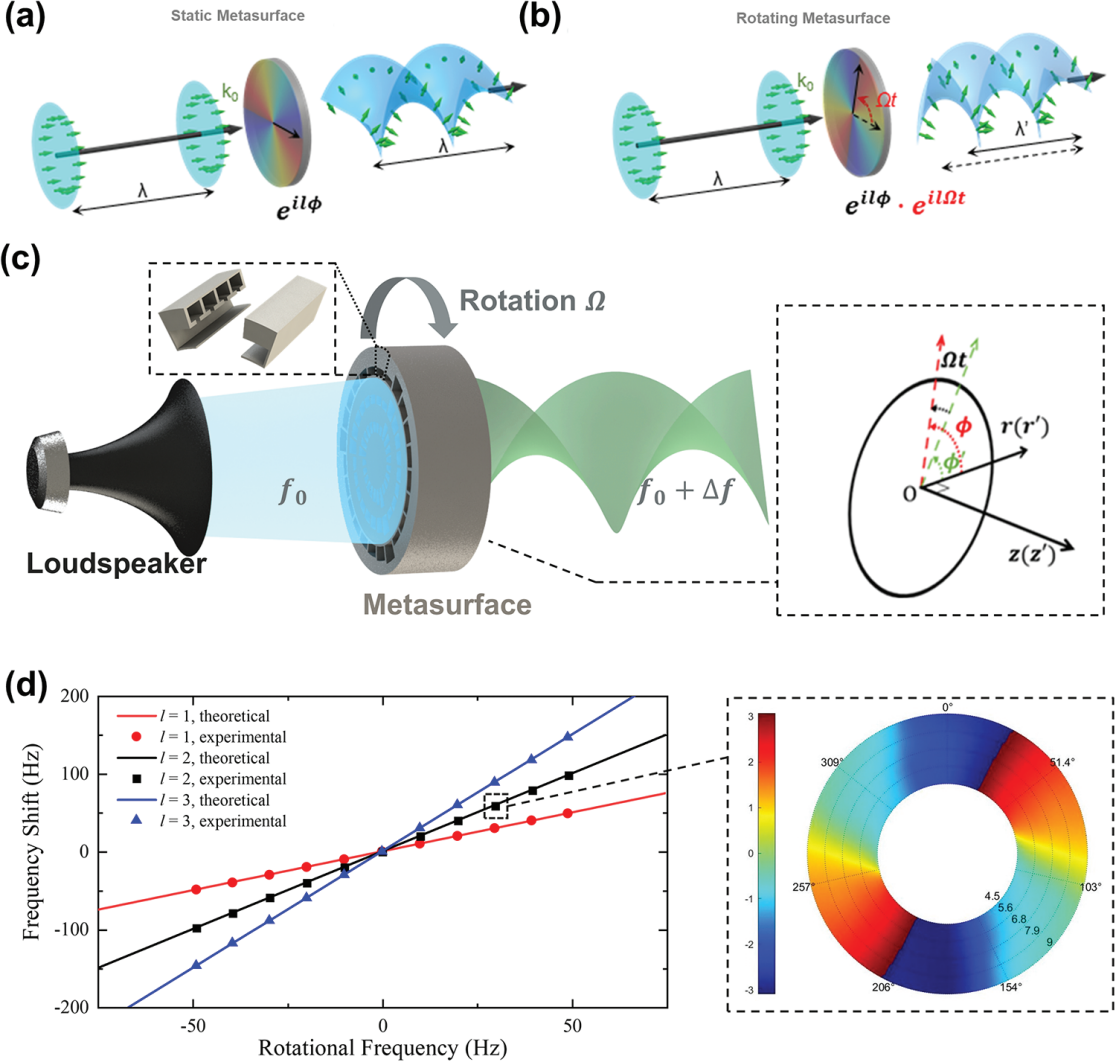
a) Schematic of generation of acoustic vortex with topological charge *l* = 2 by a static metasurface, where the sound wave is tuned to helical wavefront. b) The rotation of metasurface can twist the wavefront, resulting in an acoustic frequency shift. c) Schematic of the frequency convertor: frequency of the acoustic wave will be converted to a new frequency after passing through a specifically designed rotating metasurface. Inset (left): the 3D view of the cross section of the unit cell; and inset (right): schematic of coordinate transformation. d) Theoretical and experimental results of the frequency shift with different topological charge *l* and rotational frequency Ω/2*π*. Inset: phase distribution of the harmonic frequency at the cross section, 5.7 cm behind the metasurface with *l* = 2 and Ω/2*π* = 30 Hz.

**Figure** **1**a,b illustrates a schematic of the frequency convertor based on the rotational Doppler effect. By a plane wave incidence at the working frequency, a transmitted acoustic vortex beam with an azimuthal phase distribution expressed as *lϕ* can be produced by the metasurface with a specific topological charge *l*. The metasurface can twist the sound plane wave into a helical wavefront by adding phase factor of e^i*lϕ*^, only changing the orientation of the sound wavevector (Figure 1a). Note that the frequency of the generated acoustic vortex is equal to the fundamental frequency. When we rotate the metasurface at a frequency of Ω, the helical wavefront is further twisted or released with a phase factor of e^i*l*Ω*t*^ in the original propagating distance (Figure 1b). In this scenario, both the orientation and magnitude of sound wavevectors are changed, implying an acoustic frequency shift of Δ*f* = *l*Ω/2*π*. For a clear description, a rest frame is defined as (*r*, *ϕ*, *z*) in cylindrical coordinate, where *r* and *ϕ* are the radial and azimuthal directions of the metasurface, and *z* is the incident direction of the acoustic wave, as shown in Figure 1c. When relative motion between the acoustic wave and the metasurface appears in the azimuthal direction, the additional phase shift (*lϕ*) carried by the metasurface can generate the frequency shift (*l*Ω/2*π*) of the transmitted sound wave through the rotation of the metasurface. Assuming that a plane wave is incident vertically on a rotating metasurface, the azimuthal phase distribution of the transmitted wave can be described as *lϕ*′ in the rotational frame. Here the rotational frame referenced to the rest frame is described as (*r*′, *ϕ*′, *z*′) in cylindrical coordinates, where *r*′ = *r*, *ϕ*′ = *ϕ* − Ω*t*, and *z*′ = *z*. The plane wave can be expressed as $p_{i}=e^{i(\omega t-k_{z}z)}$ and $p_{i}^{\prime }\;=\;Ae^{i(\omega t-k_{z^{\prime }}z^{\prime })}$ in the rest and the rotational frame, respectively, where *A* is the amplitude of the plane wave. Considering the high transmittance of the metasurface, we adopt an ideal situation without scattering and loss that the transfer function of the metasurface can be expressed as $e^{-ik_{z}d}e^{il\phi }$, where *d* is the thickness of the metasurface. The expression of the transmitted wave in the rotational frame can be derived as

(1) $$p_{t}^{\prime }{|}_{z^{\prime }\;=\;d}=p_{i}^{\prime }{|}_{z^{\prime }=0}e^{-ik_{z^{\prime }}d}e^{il\phi^{\prime }}=Ae^{i\left(\omega t-k_{z}d\right)}e^{il\phi^{\prime }}$$

where *z*′ = 0 and *z*′ = *d* are the incident and transmission planes of the metasurface, respectively. The transmitted wave in the rest frame can be expressed as

(2) $$p_{t}{|}_{z\;=\;d}=p_{t}^{\prime }{|}_{z^{\prime }=d}=Ae^{i\left(\omega -l\Omega \right)t}e^{i\left(l\phi -k_{z}d\right)}$$

Hence, the frequency of transmitted wave probed by a static microphone behind the rotating metasurface can be derived as $f^{\prime }=\frac{\omega -l\Omega }{2\pi }$, and a high‐efficiency frequency convertor can be obtained by a metasurface with a high transmittance.

**Figure 2:**
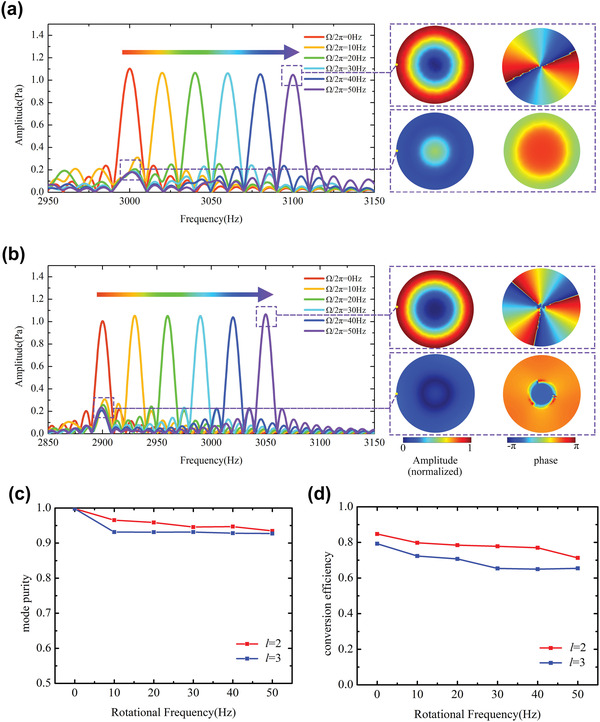
Numerical results of the frequency conversion under different rotating frequencies with topological charge *l* equal to a) 2 and b) 3. Inset: amplitude and phase distribution of the harmonic and fundamental frequency at the cross section, 5.7 cm behind the metasurface with *l* = 2 and 3, and Ω/2*π* = 50 Hz. And numerical results of the c) mode purity and d) conversion efficiency under different rotating frequency.

Series numerical simulations have been carried out to demonstrate the performance of the designed Doppler frequency convertor. **Figure** **2** demonstrates the simulated Fourier spectra of the transmitted wave behind second‐ and third‐order metasurfaces with different rotating rates, respectively. The working frequencies of the second‐ and third‐order metasurfaces are 3000 and 2900 Hz, respectively. The Fourier spectra of the transmitted acoustic waves show that the numerical results are in good agreement with the theoretical prediction. With the increase or decrease of rotation rates, the frequency of the transmitted waves will shift according to *f*_0_ + *l*Ω/2*π*, which also agrees well with the experimental results in Figure 1d. The amplitude of the main peaks in all spectra is similar to that of static case (Ω = 0), which shows that a high‐efficiency conversion of the frequency can be easily achieved by a rotating metasurface that can produce a specific order acoustic vortex. Insets in Figure 2 demonstrate the amplitude and phase distribution of the transmitted acoustic waves at the cross section, 5.7 cm behind the metasurface with the rotational frequency equal to 50 Hz. In the transmitted waves, only a small part of the acoustic energy remains in the fundamental component, and most of energy is converted into the harmonic components. The ratios of harmonic component intensity in transmitted wave to the total intensity of incident wave and transmitted wave are defined as the purity and efficiency of frequency conversion, respectively, which are above 93% and 65% in numerical simulations, as shown in Figure 2c,d. Note that the topological charge of the harmonic component in the transmitted wave is determined by the metasurface and will not be affected by the rotating speed, while the fundamental component still maintains the original topological charge of the incident wave. This means that only acoustic waves with additional phase modulation from metasurface can result in frequency conversion.

**Figure 3:**
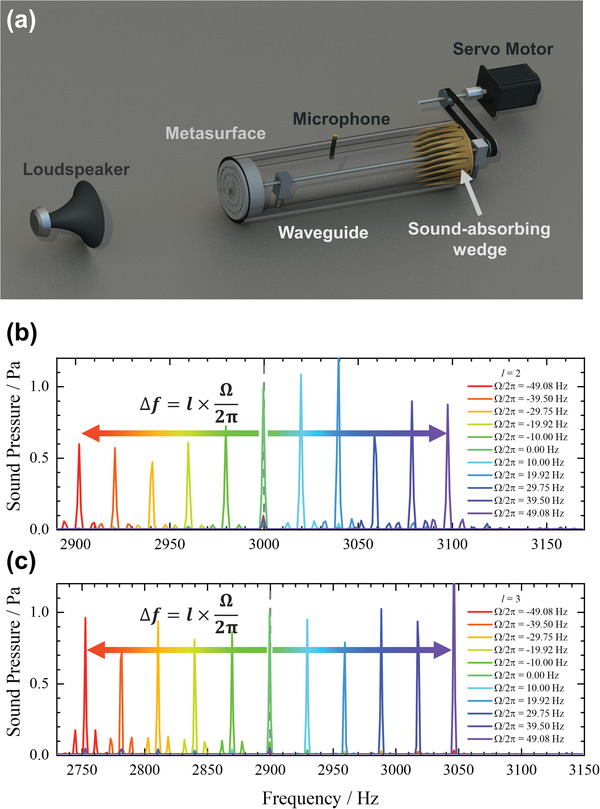
a) Experimental setup for frequency convertor. A metasurface driven by a servo motor is covered by an acoustic waveguide whose end is blocked by a wedge absorber and a microphone is placed 20 cm behind the metasurface. A bearing bracket is used to secure the drive shaft. b,c) Experimental measurement of the frequency shift under different rotational frequencies with topological charge equal to b) 2 and c) 3, respectively.

Next, we performed experiments to verify the results of the theoretical prediction and numerical simulations. **Figure** **3**a demonstrates the experimental setup for frequency conversion produced by an acoustic metasurface with different topological charge *l* and rotational frequency Ω/2*π*. Also, a measured phase distribution (Figure 1d) at the cut‐plane perpendicular to the axial direction 20 cm behind the metasurface illustrates an acoustic vortex (*l* = 2) with 60 Hz frequency shift. (Our measure plane is farther away from the metasurface than that in simulation mainly because the spiral phase distribution and the amplitude hardly change in cylindrical waveguides at different cross sections due to the high mode purity of the transmitted wave, and the probability of microphone damage increases greatly when it is too close to a rotating metasurface.) These all agree well with the results from the numerical simulations except for slight reduction in the efficiency of the frequency conversion, which results from the influence of the scattered wave produced by the bearing bracket and other essential parts in the rotating transmission system, and indicate that the rotation of the metasurface does not destroy the vortex wavefront that the metasurface should have produced in static media. Also, the fundamental frequency component in the transmitted waves mainly results from the leakage of the fundamental waves caused by the slit between the waveguide and the rotating metasurface. Nevertheless, according to the numerical simulations, above 93% purity can be achieved in the frequency convertor, which indicates that the conversion efficiency can be nearly perfect in our ideal model given the metasurface only changes the propagation phase while keeping a near‐unity transmission efficiency. The measured efficiency is slightly lower than simulation due to the imperfect impedance match between air and the experimental samples and the scattering from the necessary fixing and transmission accessories.

Figure 3b,c demonstrates the Fourier spectra of the transmitted wave behind second‐ and third‐order metasurfaces with different rotating rates, respectively. The working frequencies of the second‐ and third‐order metasurfaces are 3000 and 2900 Hz, respectively. Although the rotation of the metasurface generates airflow inevitably, the Fourier spectra of the transmitted acoustic waves show that the theoretical prediction is in good agreement with the experimental results, which indicates that the airflow has little influence on frequency conversion. In complete agreement with the results of the numerical simulations, the amplitude of the main peaks in all spectra is similar to that of static case (Ω = 0), which indicates that the experimental samples made from photopolymer can easily maintain high‐purity and high‐efficiency frequency conversion through rotating mechanisms. The peaks referring to fundamental frequency with small amplitude remain on the spectrum, which indicates that the energy of the fundamental waves almost completely converts to a new frequency through a simple rotating system. As the theoretical prediction mentioned, the frequency conversion is based on perfect spiral phase distribution or OAM mode, so that the harmonic component in the transmitted waves has perfect spiral phase distribution of OAM, which is verified numerically and experimentally in Figures 1d and 2. However, the fundamental component cannot obtain OAM from the metasurface and keeps the original wave front without spiral phase distribution, as shown in the insets of Figure 2. Furthermore, owing to the additional spiral phase distribution of e^i*lϕ*^ and efficiency of the frequency conversion, the convertor is potential to serve as a frequency‐tunable OAM generator and more versatile wave manipulation, such as the frequency modulator.

**Figure 4:**
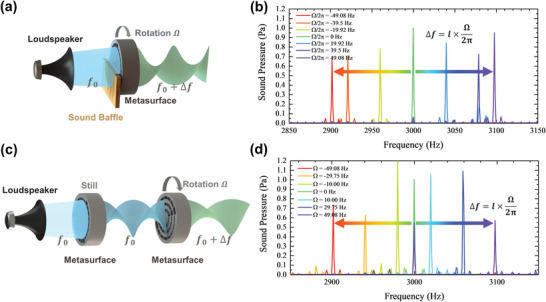
a) Schematic of a frequency convertor with the metasurface being half covered; the obstacles can be selected arbitrarily. b) Experimental measurement of the frequency shift under different rotational frequencies with topological charge being equal to 2 and fundamental frequency being equal to 3000 Hz, corresponding to the experimental conditions shown in panel (a). c) Schematic of rotational Doppler frequency shift with spiral waves being incident; a static metasurface is used as a spiral wave emitter. d) Experimental measurement of the frequency shift under different rotational frequency with topological charge being equal to 2 and fundamental frequency being equal to 3000 Hz, corresponding to the experimental conditions shown in panel (c).

Furthermore, the proposed mechanism is insensitive to the shape of incident wavefront. The frequency convertor half occluded by an obstacle can still work efficiently in our experiment, as shown in **Figure** **4**a. Behind the metasurface, a perfect acoustic vortex cannot be formed because of the absence of the acoustic waves transmitted from half of the blocked area, but the efficient frequency shift is still verified experimentally, as shown in Figure 4b, which can be ascribed to the robustness to the incident wavefront. Moreover, after a static metasurface utilized to produce a specific order acoustic vortex wave, the rotating metasurface can still shift the frequency robustly, as shown in Figure 4c. The Fourier spectra of the transmitted wave under different rotating rates prove that the conversion efficiency is robust for different rotating rates, which changes the magnitude of frequency shift (Figure 4d). These results indicate that an efficient cascade scheme of the frequency convertor can be realized to amplify the frequency shift.

**Figure 5:**
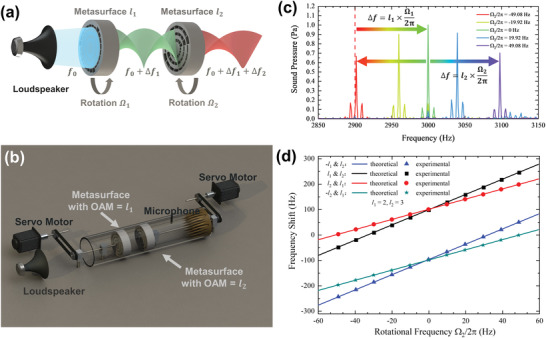
a) Schematic of cascade scheme of the frequency convertor: a metasurface with topological charge being equal to *l*_1_ rotating at Ω_1_ is cascaded with another metasurface topological charge being equal to *l*_2_ rotating at Ω_2_. b) Schematic of experimental device for the cascade scheme in panel (a). c) Experimental measurement of the cascaded frequency conversion under different rotational frequencies Ω_2_ with Ω_1_ = −2*π* × 49.08 Hz, *l*_1_ = −2 and *l*_2_ = 2; the fundamental frequency and the working frequency of the metasurface *l*_2_ are equal to 2900 and 3000 Hz, respectively. d) Theoretical prediction and experimental measurements of the cascaded frequency shift with different topological charges *l_i_* and rotational frequency Ω_*i*_/2*π*, *i* = 1, 2; Ω_2_/2*π* = 0 correspond to the frequency shift from the metasurface *l*_1_ before cascading.

**Figure** **5**a explains the mechanism of frequency conversion in such a system consisting of two cascading metasurfaces with topological charges chosen as *l*_1_ and *l*_2_, respectively, and what happens when an incident wave passes through these two layers. Each metasurface with its respective topological charge and rotating speed will give rise to an extra frequency shift Δ*f_i_* to the transmitted wave passing through it and the frequency of the output beam will be converted into $f_{0}+\sum_{i}^{N}\Delta f_{i}$, where *N* = 2 is the number of the metasurfaces and Δ*f_i_* = Ω_*i*_ *l_i_*/2*π* with the subscript *i* corresponding to the sequence of the metasurfaces in the cascade scheme. Illuminated by the incident acoustic wave with a frequency of *f*_0_ being equal to the working frequency of the first metasurface *l*_1_, the first frequency convertor twists the wave front of the incident plane wave into a vortex with the topological charge being equal to *l*_1_ and converts the frequency of the wave to *f*_0_ + Δ*f*_1_. When the new frequency meets with the working frequency of the next metasurface *l*_2_, a further frequency conversion happens with the further frequency shift and additional topological charge being equal to Δ*f*_2_ and *l*_2_. As shown in Figure 5d, theoretical predictions and experimental measurements are in good agreement with different frequency manipulations by a cascade scheme of the frequency convertor. The frequency of the acoustic wave passing through the first frequency convertor can be further manipulated in the frequency domain. The topological charge and rotational angular velocity are tunable, which endow a lot of freedom for frequency manipulation. Furthermore, when $\sum_{i}^{N}l_{i}=0$, the process of topological charge generation and frequency conversion can be separated; as a simplest example, two cascaded metasurfaces with topological charges of +2 and −2, respectively. Figure 5c verifies the efficiency of the cascade scheme with different angular velocities of the second metasurface *l*_2_. There is almost no component for fundamental frequency in the spectrum of the transmitted waves in Figure 5c, indicating a high‐purity conversion still maintained in the cascade scheme. Also all the amplitudes of the shifted frequencies at different angular velocities are similar to that of the static one (Ω_2_ = 0 rad s^−1^), verifying the high efficiency of the cascading scheme.

## 3 Conclusions

In summary, we theoretically and experimentally present the design of a frequency convertor for efficiently converting the frequency of acoustic wave with metasurfaces that themselves do not serve as active sources for emitting sound waves. After passing through the frequency convertor, the incident acoustic waves can generate a vortex beam with specific additional OAM from the metasurface. Different from nonlinear media, our mechanism can generate harmonic frequencies that are not integer multiples of the fundamental frequency and the proportion of the harmonic component of transmitted waves does not change with the amplitude of the incident wave. The frequency conversion mechanism can be extended by cascading several frequency convertors to achieve a high‐efficiency frequency shift, verified by cascading two frequency convertors in our experiments. The designed frequency convertor and its cascade scheme provide new approaches of manipulating acoustic waves in frequency domain. Such a rotating metasurface can efficiently generate acoustic vortex beams with stable topological charges at varying frequencies, which may significantly impact the future acoustic communications. The precise correspondence between frequency shift and rotating rates enables our design to measure the actual rotating rates of an object. Our design is also applicable to other types of metasurfaces as long as their impedances are not severely mismatched to the background medium, and can be equivalent to a time‐varying metamaterial due to the rotation. The freedom of manipulation is greatly expanded by combining with background media with tunable refractive index,^[^ ^35^, ^36^ ^]^ offering the possibility of finding more extensive applications in noise control, biomedical applications, and ultrasound control, etc.

## 4 Experimental Section

Numerical simulations were carried out in COMSOL Multiphysics using the transient pressure acoustic module and rotating domain. The integral of the transmitted sound intensity of harmonic frequency was calculated numerically to evaluate the efficiency and purity. The geometry used in the simulation was the same as that in the experiment, but the scattering caused by fixed equipment was neglected for simplicity. The data length of the numerical simulation results was limited, resulting in low spectral resolution, but this would not hinder the accuracy of the conclusion.

Figures 3a and 5b demonstrate the experimental systems for the frequency convertor and the cascade scheme. A metasurface made of photopolymer was driven by a servo motor via a transmission shaft and tooth synchronous belt. The bearing system fixed to the vibration isolation platform was used to ensure smooth rotation of the shaft without axial slip. Acoustic waves generated from a compression driver with a horn were incident on the rotating metasurface, which was covered by a waveguide to better mimic a plane wave and avoid the influence of the diffraction of the incident waves. The wedge made of sound‐absorbing foam was placed at the end of waveguide to eliminate the undesired reflection. The radius of the metasurface used in the frequency convertor was 9.15 cm, which was about 0.8 wavelength of the working frequency. All the metasurfaces were fabricated by 3D printing technology and fixed with the shaft through flanges. The design of the metasurfaces is demonstrated in the Supporting Information. There were some unavoidable vibrations of the rotating metasurfaces caused by centrifugal action in the experiments so that the radius of the waveguide, set as 9.5 cm, needed to be slightly larger than that of the metasurfaces. The 0.25 in. Brüel and Kjær type‐4961 microphones were used to measure the sound pressure distribution behind the rotating metasurface in the waveguide. An additional microphone of the same type was used as the reference microphone in the measurement of the phase distribution of transmitted waves. Servo motors provided the rotating power for the metasurfaces through the gears and belts with a speed ratio of 1:1. In order to eliminate the error between the actual rotating speed and the number displayed on the servo motor controller, the speed in all experiments was calibrated by a tachometer whose error was less than 1/60 Hz.

## Conflict of Interest

The authors declare no conflict of interest.

## Supporting information

Supporting Information

## Data Availability

The data that support the findings of this study are available from the corresponding author upon reasonable request.

## References

[1] R. W. Boyd , Nonlinear Optics, Elsevier Science, Amsterdam, Netherlands 2020.

[2] B. S. Richards , D. Hudry , D. Busko , A. Turshatov , I. A. Howard , Chem. Rev. 2021, 121, 9165. 34327987 10.1021/acs.chemrev.1c00034

[3] G. Gui , N. J. Brooks , H. C. Kapteyn , M. M. Murnane , C. T. Liao , Nat. Photonics 2021, 15, 608.

[4] S. Y. Emelianov , M. F. Hamilton , Y. A. Ilinskii , E. A. Zabolotskaya , J. Acoust. Soc. Am. 2004, 115, 581. 15000170 10.1121/1.1621858

[5] B. Liang , X. Y. Zou , J. C. Cheng , J. Acoust. Soc. Am. 2008, 124, 1419. 19045634 10.1121/1.2957931

[6] A. M. Yao , M. J. Padgett , Adv. Opt. Photonics 2011, 3, 161.

[7] X. Jiang , J. J. Zhao , S. L. Liu , B. Liang , X. Y. Zou , J. Yang , C. W. Qiu , J. C. Cheng , Appl. Phys. Lett. 2016, 108, 203501.

[8] L. Allen , M. W. Beijersbergen , R. J. C. Spreeuw , J. P. Woerdman , Phys. Rev. A 1992, 45, 8185. 9906912 10.1103/physreva.45.8185

[9] J. C. Ni , C. Huang , L. M. Zhou , M. Gu , Q. H. Song , Y. Kivshar , C. W. Qiu , Science 2021, 374, 418. 10.1126/science.abj0039 34672745

[10] M. J. Padgett , J. Opt. A: Pure Appl. Opt. 2004, 6, S263.

[11] K. D. Skeldon , C. Wilson , M. Edgar , M. J. Padgett , New J. Phys. 2008, 10, 013018.

[12] C. E. M. Demore , Z. Y. Yang , A. Volovick , S. Cochran , M. P. MacDonald , G. C. Spalding , Phys. Rev. Lett. 2012, 108, 194301. 23003045 10.1103/PhysRevLett.108.194301

[13] G. X. Li , T. Zentgraf , S. Zhang , Nat. Phys. 2016, 12, 736.

[14] J. C. Ni , S. L. Liu , D. Wu , Z. X. Lao , Z. Y. Wang , K. Huang , S. Y. Ji , J. W. Li , Z. X. Huang , Q. H. Xiong , Y. L. Hu , J. R. Chu , C. W. Qiu , Proc. Natl. Acad. Sci. USA 2021, 118, e2020055118.33372145

[15] D. G. Grier , Nature 2003, 424, 810. 12917694 10.1038/nature01935

[16] J. C. Ni , C. W. Wang , C. C. Zhang , Y. L. Hu , L. Yang , Z. X. Lao , B. Xu , J. W. Li , D. Wu , J. R. Chu , Light: Sci. Appl. 2017, 6, e17011. 30167269 10.1038/lsa.2017.11 PMC6062222

[17] K. I. Willig , S. O. Rizzoli , V. Westphal , R. Jahn , S. W. Hell , Nature 2006, 440, 935. 16612384 10.1038/nature04592

[18] J. Wang , J. Y. Yang , I. M. Fazal , N. Ahmed , Y. Yan , H. Huang , Y. X. Ren , Y. Yue , S. Dolinar , M. Tur , A. E. Willner , Nat. Photonics 2012, 6, 488.

[19] Z. F. Zhang , X. D. Qiao , B. Midya , K. Liu , J. B. Sun , T. W. Wu , W. J. Liu , R. Agarwal , J. M. Jornet , S. Longhi , N. M. Litchinitser , L. Feng , Science 2020, 368, 760. 32409473 10.1126/science.aba8996

[20] C. Z. Shi , M. Dubois , Y. Wang , X. Zhang , Proc. Natl. Acad. Sci. USA 2017, 114, 7250. 28652341 10.1073/pnas.1704450114 PMC5514747

[21] X. Jiang , B. Liang , J. C. Cheng , C. W. Qiu , Adv. Mater. 2018, 30, 1800257. 10.1002/adma.201800257 29602184

[22] B. Y. Liu , H. Giddens , Y. Li , Y. J. He , S. W. Wong , Y. Hao , Opt. Express 2020, 28, 3745. 32122036 10.1364/OE.382700

[23] H. L. Zhou , D. Z. Fu , J. J. Dong , P. Zhang , X. L. Zhang , Opt. Express 2016, 24, 10050. 27137615 10.1364/OE.24.010050

[24] P. Georgi , C. Schlickriede , G. X. Li , S. Zhang , T. Zentgraf , Optica 2017, 4, 1000.

[25] L. Allen , M. Babiker , W. L. Power , Opt. Commun. 1994, 112, 141.

[26] S. Barreiro , J. W. R. Tabosa , H. Failache , A. Lezama , Phys. Rev. Lett. 2006, 97, 113601. 17025882 10.1103/PhysRevLett.97.113601

[27] J. Courtial , K. Dholakia , D. A. Robertson , L. Allen , M. J. Padgett , Phys. Rev. Lett. 1998, 80, 3217.

[28] I. Bialynicki‐Birula , Z. Bialynicki‐Birula , Phys. Rev. Lett. 1997, 78, 2539.

[29] J. Courtial , D. A. Robertson , K. Dholakia , L. Allen , M. J. Padgett , Phys. Rev. Lett. 1998, 81, 4828.

[30] B. Y. Liu , H. C. Chu , H. Giddens , R. L. Li , Y. Hao , Stem Cells Int. 2019, 9, 8971.

[31] M. P. J. Lavery , F. C. Speirits , S. M. Barnett , M. J. Padgett , Science 2013, 341, 537. 23908234 10.1126/science.1239936

[32] X. Jiang , Y. Li , B. Liang , J. C. Cheng , L. K. Zhang , Phys. Rev. Lett. 2016, 117, 034301. 27472113 10.1103/PhysRevLett.117.034301

[33] Z. Y. Guo , H. J. Liu , H. Zhou , K. Y. Zhou , S. M. Wang , F. Shen , Y. B. Gong , J. Gao , S. T. Liu , K. Guo , Phys. Rev. E 2019, 100, 053315. 31870012 10.1103/PhysRevE.100.053315

[34] G. M. Gibson , E. Toninelli , S. A. R. Horsley , G. C. Spalding , E. Hendry , D. B. Phillips , M. J. Padgett , Proc. Natl. Acad. Sci. USA 2018, 115, 3800. 29581257 10.1073/pnas.1720776115 PMC5899465

[35] Z. X. Liang , J. S. Li , Phys. Rev. Lett. 2012, 108, 114301. 22540476 10.1103/PhysRevLett.108.114301

[36] C. Q. Xu , G. C. Ma , G. Chen , J. Luo , J. J. Shi , Y. Lai , Y. Wu , Phys. Rev. Lett. 2020, 124, 074501. 32142328 10.1103/PhysRevLett.124.074501

