# Supplementary material for: Efficient and High‐Purity Sound Frequency Conversion with a Passive Linear Metasurface
Source: Adv Sci (Weinh). 2022 Oct 17;9(33):2203482. doi: 10.1002/advs.202203482 (PMC9685439; doi:10.1002/advs.202203482)
Supplement: Supplementary file 1 — Supporting Information [file ADVS-9-2203482-s001.pdf]

ADVANCED SCIENCE
Open Access

Supporting Information

for *Adv. Sci.*, DOI 10.1002/advs.202203482

Efficient and High-Purity Sound Frequency Conversion with a Passive Linear Metasurface

Wei Wang, Chengbo Hu, Jincheng Ni, Yujiang Ding, Jingkai Weng, Bin Liang, Cheng-Wei Qiu* and Jian-Chun Cheng**

Supporting Information

S1. The design of acoustic metasurface for frequency convertor

In the current study, we implement the metasurface in the frequency convertor by using a specific kind of acoustic metamaterial unit cell composed of four local resonators and a straight pipe, as shown in **Figure S1**a. The length of the pipe, $L = \lambda/2$, is half of wavelength of the working frequency and the dimensions of four local resonators are exactly the same with an adjustable parameter, h_1, which is the height of the cavity. In Figure S1b, the designed metasurface is assembled from three layers consisting of $8l$ fanlike sections with different h_1, where l is the topological charge of the metasurface. The radius of the metasurface is $R = 4h_0$ and the holes in the centre of the metasurface are reserved for mounting to the transmission shaft. In this design, the series connection of cavities supports strong local resonance for effectively slowing down the propagation of wave as it passes by, enabling free control of the propagation phase within the full 0-to-2π range. On the other hand, the impedance mismatch caused by these local resonators is maximally compensated by the introduction of the straight pipe supporting Fabry-Perot resonance, which results in hybrid resonance and ensures full 2π control of phase shift while keeping a near-unity transmission efficiency. This is verified by the numerical results depicted in Figure S1c, which shows that the phase shift can be smoothly tuned within the range from 0 to 2π by adjusting a single structural parameter, h_1 ,which is the height of cavities, with no need of changing the overall size of each subwavelength unit cell ($L = \lambda/2$ and $h_0 = \lambda/5$). The phase delay and the transmissibility as functions of h_1 of the fanlike unit cell for different layers need to be numerically calculated respectively, and the acoustic pressure transmittance of every cavity assembled in the metasurface needs to be larger than 0.8. The acoustic intensity transmittance of the whole metasurface is around 0.77.

It is also noticed that such a metamaterial design has a rotationally symmetric structure and a thickness only half of wavelength, which allows the metasurface to be easily driven by the transmission system and rotate at high speeds without the violent vibration caused by the counterweight imbalance.

S2. Additional Discussion

The centre area of metasurface used in the frequency convertor is reserved for install the flange which can be fixed on the transmission shaft through jack screws, as shown in **Figure S2**a. As a matter of convenience, the waveguides, in Figures S2b and c, are assembled with perfectly cut pieces and the ends of those are filled with sound-absorbing sponge wedges that prevent the reflection of sound waves. The loudspeaker is directly placed on the table top of the shock absorption platform and radiates sound waves at 3000 Hz to the mouth of the waveguide, which resulting the scattering of the incident wave from the desk top and the nonuniformity of acoustic pressure amplitude distribution of the incident wave. In order to avoid the collision between rotating metasurface and the waveguide, the radii of the waveguide and the metasurface are set to be 9.50 cm and 9.15cm, which leaves a slit that can be a tunnel for the fundamental wave inevitably. Furthermore, for the stable operation of the transmission system, fixed components (mainly bearings and bearing brackets) are essential but a big scatterer for incident waves in the waveguide, as shown in Figure S2c.

It is also noticed that the same direction of rotation of the cascade frequency convertor is also a significant influential factor for the efficiency of the cascade scheme, in which case air between two rotating metasurface will rotate in the same direction due to the disturbance of two metasurfaces in the closed space between two metasurfaces in particular, which will weaken the rotational Doppler effect. Also, axial vibration is common in the experiments that results in the side lobes close to the main peak of the transmitted waves in the spectrum.

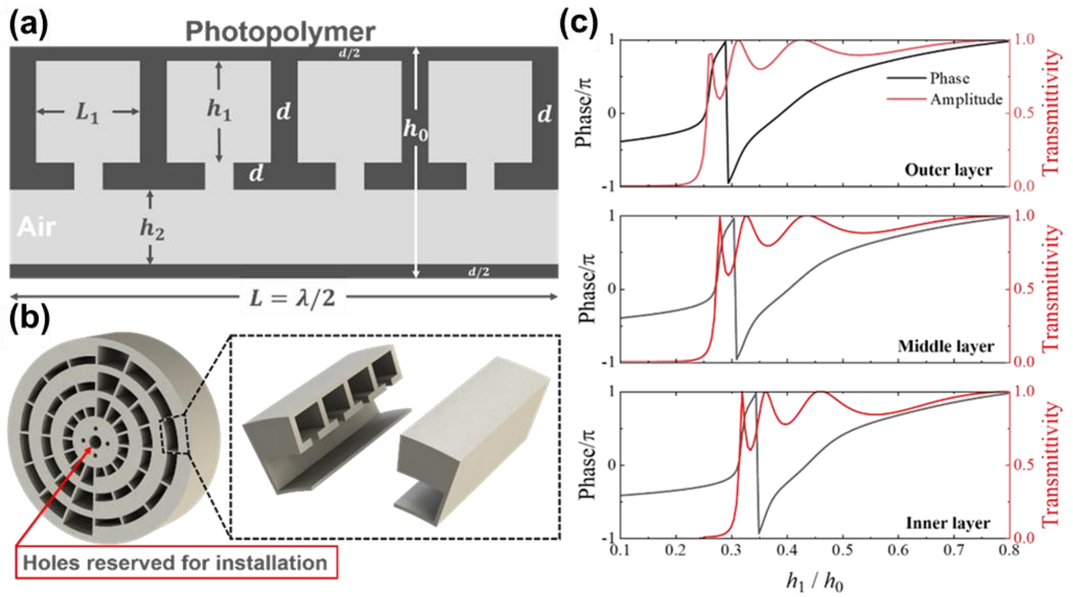

Figure S1. Acoustic metasurface (OAM equal to 2 for example), basic building block of the frequency convertor, is formed by coupling four local resonators and a straight pipe for producing a coupled resonance that compensates the impedance mismatch in the phase modulation. a) 2D schematic view of an individual unit cell with adjustable geometry parameters; b) 3D schematic of the designed metasurface which is divided into three layers along the radius (inner layer, middle layer, and outer layer from inside to outside). Inset: the 3D view of the cross-section of the unit cell; c) The simulated phase discontinuity provided by this unit cell as a function of the height of cavity, h_1, shows that adjustment of this single parameter ensures full 2π phase control while keeping near-unity transmission efficiency.

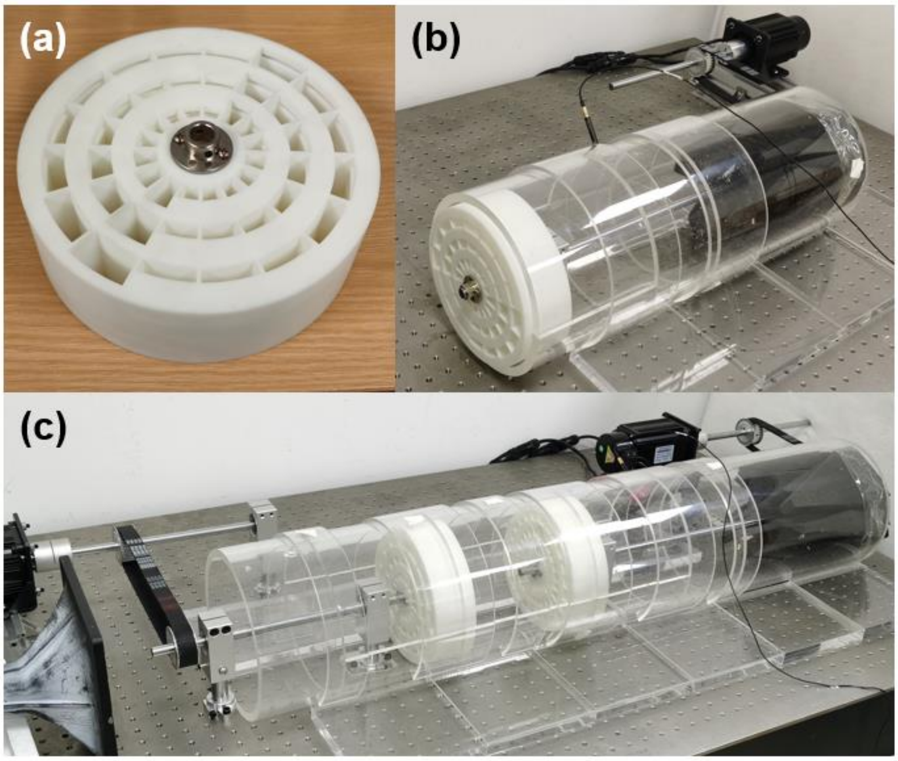

Figure S2. a) Photo of 3D printed acoustic metasurface prototype with topological charge equal to 2; (b, c) Experimental setup for (b) frequency convertor: a flange is used to secure the metasurface to the shaft driven by a servo motor and (c) cascade scheme: each metasurface is driven by a servo motor respectively.
